# Modified Protocol to Enable the Study of Hemorrhage and Hematoma in a Traumatic Brain Injury Mouse Model

**DOI:** 10.3389/fneur.2021.717513

**Published:** 2021-09-28

**Authors:** Hyejin Joo, Jinhyun Bae, Jae-Woo Park, Beom-Joon Lee, Byoung Dae Lee, Youngmin Bu

**Affiliations:** ^1^Department of Science in Korean Medicine, Graduate School, Kyung Hee University, Seoul, South Korea; ^2^Department of Herbal Pharmacology, College of Korean Medicine, Kyung Hee University, Seoul, South Korea; ^3^Department of Internal Medicine, College of Korean Medicine, Kyung Hee University, Seoul, South Korea; ^4^Department of Physiology, Kyung Hee University School of Medicine, Seoul, South Korea

**Keywords:** traumatic brain injury, controlled cortical impact, hemorrhage, mouse, protocol

## Abstract

To date, many studies using the controlled cortical impact (CCI) mouse model of traumatic brain injury (TBI) have presented results without presenting the pathophysiology of the injury-core itself or the temporal features of hemorrhage (Hrr). This might be owing to the removal of the injury-core during the histological procedure. We therefore developed a modified protocol to preserve the injury-core. The heads of mice were obtained after perfusion and were post-fixed. The brains were then harvested, retaining the ipsilateral skull bone; these were post-fixed again and sliced using a cryocut. To validate the utility of the procedure, the temporal pattern of Hrr depending on the impacting depth was analyzed. CCI-TBI was induced at the following depths: 1.5 mm (mild Hrr), 2.5 mm (moderate Hrr), and 3.5 mm (severe Hrr). A pharmacological study was also conducted using hemodynamic agents such as warfarin (2 mg/kg) and coagulation factor VIIa (Coa-VIIa, 1 mg/kg). The current protocol enabled the visual observation of the Hrr until 7 days. Hrr peaked at 1–3 days and then decreased to the normal range on the seventh day. It expanded from the affected cortex (mild) to the periphery of the hippocampus (moderate) and the brain ventricle (severe). Pharmacological studies showed that warfarin pre-treatment produced a massively increased Hrr, concurrent with the highest mortality rate and brain injury. Coa-VIIa reduced the side effects of warfarin. Therefore, these results suggest that the current method might be suitable to conduct studies on hemorrhage, hematoma, and the injury-core in experiments using the CCI-TBI mouse model.

## Introduction

Traumatic brain injury (TBI) is composed of complex pathological processes in the brain, including physical brain damage, hemorrhage (Hrr), hematoma, and ischemic injury after mechanical trauma (1). Generally, TBI can be divided primarily as a closed or penetrative head injury based on the absence or presence of skull damage. Closed head injuries are more frequent but mild and tend to heal spontaneously. Penetrative head injuries are infrequent but are often life-threatening. In addition, it can cause various cerebral Hrr or hematomas that worsen brain damage and cause serious complications (2). Concerning the pathophysiology of penetrative head injuries, mechanical force-induced brain contusion and Hrr are followed by the formation of a hematoma at the early stage (3, 4). Hrr and hematoma are regarded as important pathologic factors to be evaluated as the cause of death in patients with invasive brain damage (5, 6). Hrr in the brain parenchyma following TBI could be a space-occupied mass and thus could induce the compressive pressure to local tissue followed by a reduction of blood flow and intracranial pressure (ICP) elevation. In addition, recent studies have shown that anticoagulant therapy, including warfarin, worsens the symptoms rapidly in patients with TBI due to side effects such as massive Hrr (7). Therefore, therapeutic management of Hrr or hematomas might be a key point in the treatment of TBI, which is a common procedure in clinics.

**Figure 1:**
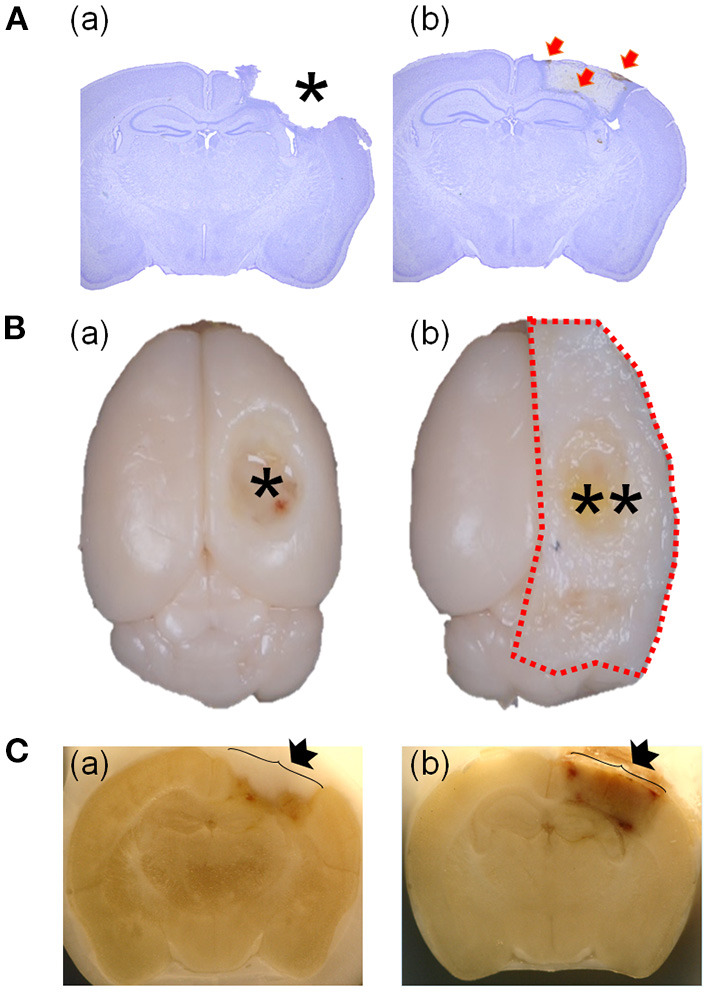
Comparison of the representative brain using the previous protocol with a missing injured area and the representative brain using the current protocol with preserving the injured area. Brains were stained at 7 days after TBI (2.5 mm of depth). The asterisk indicates the empty area where the injured area is located **(Aa)**. The weakly stained area might be the injured area **(Ab)**. Red arrows indicate the hematoma particles around the injured area **(Ab)**. Comparison of brains isolated with **(Ba)** or without **(Bb)** skull bone and a photograph taken during the brain slicing procedure **(C)** with **(Ca)** or without **(Cb)**. The isolated brain without a skull bone shows the empty space in the injured area (asterisk in **Ba**) and another with a skull bone (two asterisks in **Bb**). Image of the side of −1.82 mm from the bregma of a brain in the course of brain slicing with cryocut **(C)**. The arrow indicates the skull bone covering **(Cb)** or without covering **(Ca)** or the injured area. TBI, traumatic brain injury.

Various TBI animal models have been developed and are well-documented (8). Despite the disadvantages of invasive craniectomy (9–11), the controlled cortical impact (CCI)-TBI model has been regarded as an excellent model of penetrative head injury for >20 years because of its convenience, reproducibility, and the similarity of clinical features, including brain injury and functional deficits (8). Despite the importance of Hrr and hematoma in penetrative head injury, most studies using the CCI-TBI model have not investigated the temporal features of Hrr, hematoma, and brain injury and have presented results with a missing injury-core (Figure 1Aa). Even if there is research on TBI Hrr, only the Hrr pattern is reported 1–2 days after TBI induction (12), which is limited to studies that estimate the Hrr volume by measuring the amount of hemoglobin. Most importantly, the missing injury-core is likely to occur in the course of brain isolation procedures (Figure 1B). In the current study, we therefore developed a new protocol for preserving the injury-core (Figure 1C) by changing some steps in the histological procedure in the CCI-TBI mouse model, enabling the observation of the pathology in the injury-core, the temporal features Hrr, and hematoma. In addition, we also conducted a pharmacological study by administering warfarin (an anticoagulant agent) and injecting coagulation factor VIIa (Coa VIIa, inhibiting Hrr) to evaluate whether the protocols were as appropriate as those for the Hrr study in mice with TBI.

## Methods

### Animals

All surgical procedures were approved by the Kyung Hee University Institutional Animal Care and Use Committee (KHUASP(SE)-20-132). Institute of Cancer Research (ICR) mice were purchased from Daehan Biolink (Umsung, Korea) and acclimatized for 1 week. The animals were housed under controlled temperature conditions (22°C ± 2°C), with constant humidity and a 12-h light/dark cycle. Food and water were provided *ad libitum*.

### Traumatic Brain Injury Induction

TBI was induced by the modified method based on previous studies (11, 13–15). Mice (25–30 g) were placed on a stereotaxic apparatus under anesthesia with isoflurane (1.5%). A circular craniotomy (4 mm) was performed on the right hemisphere using an electric drill (−2 mm anteroposterior and 2 mm mediolateral to the bregma). CCI was induced with an electric impact device (Leica Microsystems, Wetzlar, Germany) using a rounded impact tip (2.5 mm) at a velocity of 2 m/s at various depths (1.5–3.5 mm) and a duration of 300 ms after tilting 15° laterally. For pharmacological studies, 2.5-mm depth CCI-TBI was used. The surgical site was re-covered by the skull after surgery. The body temperature was controlled at 37°C ± 0.5°C. The sham animals underwent craniotomy without any impact.

### Grouping and Drug Administration

To analyze the relationship between severity and Hrr pattern, mice were divided into three groups: TBI depth 1.5 mm group (mild Hrr), TBI depth 2.5 mm group (moderate Hrr), and TBI depth 3.5 mm group (severe Hrr) (*n* = 6, respectively). Mice were randomly divided into three groups for pharmacological study: TBI group; TBI + warfarin (Warfarin) group; and TBI + warfarin + coagulation factor (Warfarin + Coa) group (*n* = 5–6). Warfarin [5-mg Coumadin tablet [warfarin sodium, Sigma-Aldrich, St. Louis, MO, USA]] was dissolved in 375 ml water and administered by oral uptake through bottled drinking water for 24 h before TBI induction. This dosage corresponds to a warfarin uptake of 0.08 mg (2 mg/kg) per mouse over 24 h (16). Human recombinant coagulation factor VIIa (1 mg/kg; dissolved in histidine diluent, NovoSeven; Novo Nordisk, Bagsværd, Denmark) was injected via the tail vein immediately after CCI. The TBI group received the same volume of water.

### Brain Isolation and Slicing

For histological studies, mice were anesthetized with urethane (1.2 g/kg, i.p., Daejung Chemicals & Metals Co., Ltd., Siheung-si, South Korea) and perfused transcardially with 20 ml of 0.5% heparinized saline followed by 20 ml of 4% paraformaldehyde [PFA; in 0.1 M of phosphate buffer [PB]] on the appointed day. Modifications to the protocol were performed for brain isolation and brain slicing procedures. Briefly, the mouse head was decapitated and post-fixed in 4% PFA (25 ml) for 24 h at 4°C. Then, isolation of the brain was conducted by removing the skull bone with or without the ipsilateral skull bone after incision of the midline of the skull (Figure 1B). The isolated brain, with the ipsilateral skull bone, was post-fixed once again in 4% PFA (20 ml) for 24 h at 4°C. The isolated brain was placed in 30% sucrose for 48 h at 4°C and then sliced into sections of 40-μm thickness using a cryocut (Carl Zeiss, Oberkochen, Germany; Figure 1C, where black arrow indicates with (Cb) or without (Ca) skull bone). During the brain slicing procedure, any damage to the blade, due to cutting of the bone-containing tissue, might cause subsequent damage to histology samples. It is important to note that a new side of the blade should be used to cut each brain, and the blade must be replaced after every four brains to obtain clean histology samples. To observe the temporal and spatial features of Hrr and hematoma, the side of the midline of the impacting [−1.82 mm from the bregma (17)] of each brain [1, 3, 5, and 7 dpi (days post-induction)] was photographed with a camera (Canon, Tokyo, Japan) in the middle of the brain slicing procedure (Figure 1C).

### Histological Staining

Brain slices were stained with cresyl violet (CV; Sigma, USA). Brain slices were placed on a glass slide and placed in a jar filled with CV for 3 min and subsequently dehydrated through immersion in serial ethanol baths of 50, 70, 90, and 100% alcohol. Xylene was used to clear the sections, and coverslips were then placed on slides using Permount permanent mounting medium (Fisher Chemical, Thermo Fisher Scientific, Waltham, MA, USA).

### Measuring Hemorrhagic Blood Volume by Quantitative Hemoglobin Content Determination

Mice were perfused transcardially with 40 ml of 0.5% heparinized normal saline followed by 40 ml of normal saline at 1, 3, 5, and 7 days after TBI induction. The ipsilateral hemisphere with the skull bone was isolated and homogenized using a Bullet Blender Tissue Homogenizer (Next Advance, Troy, NY, USA) in 3 ml of phosphate-buffered saline (PBS) and then centrifuged at 13,000 rpm for 30 min. The supernatant (250 μl) was mixed with 1,000 μl of Drabkin's reagent. The absorbance was determined with a Spectra Shell Microplate Reader reading at 540 nm, and hemorrhagic blood volumes were calculated for the entire brain (both hemispheres).

### Immunohistochemistry

Free-floating sections (30 μm, the side of −1.82 from the bregma) of TBI (2.5-mm depth, 7 dpi) were incubated with a goat anti-IBA-1 antibody (diluted 1:1,000; Abcam, Cambridge, UK) for 1 h after being blocked with 1% bovine serum albumin (BSA) PBS on the first day. On the second day, sections were washed and incubated with a biotinylated horse anti-goat IgG antibody (diluted 1:200; Vector Labs, Burlingame, CA, USA) for 60 min at room temperature. Sections were then reacted with an avidin–biotin–peroxidase complex kit (diluted 1:50, Vector Labs) at room temperature for 60 min and visualized with 0.05% 3,3′-diaminobenzidine (DAB; Sigma, USA) and 0.02% hydrogen peroxide. The stained sections were photographed under a microscope (BX51, Olympus, Tokyo, Japan).

### Statistical Analysis

Data were collected from animals that survived more than each time point (1, 3, 5, and 7 days after TBI) after removing data from animals that died within the time points. The total number of animals in the severity study was 72 mice (*n* = 6 per each time point of each group) and that in the pharmacological study was 42 mice in TBI group (1/43: dead/total); 41 mice in the Warfarin group (14/56: dead/total); and 41 mice in the Warfarin + Coa group (2/43: dead/total). Data were presented as mean ± standard error of the mean (*n* = 5–6 per each time point of each group). Statistical differences between groups were analyzed by one-way ANOVA followed by Tukey's *post-hoc* tests using GraphPad Prism v.5.0 (GraphPad software). *p* < 0.05 was considered statistically significant.

## Results

### Comparison of Histological Injury in Previous and Modified Methods

The injury-core area is an impact-induced brain lesion that is lost during the histological procedure (asterisk in Figure 1Ca). However, the injury-core area was preserved and clearly visible (low CV intensity area) in the brain tissue at 7 dpi, as assessed by the current method (Figure 1A). In addition, hematoma particles remained around the brain legion (Figure 1A, red arrows).

### Comparison of Hemorrhage Related to the Traumatic Brain Injury Severity

**Figure 2:**
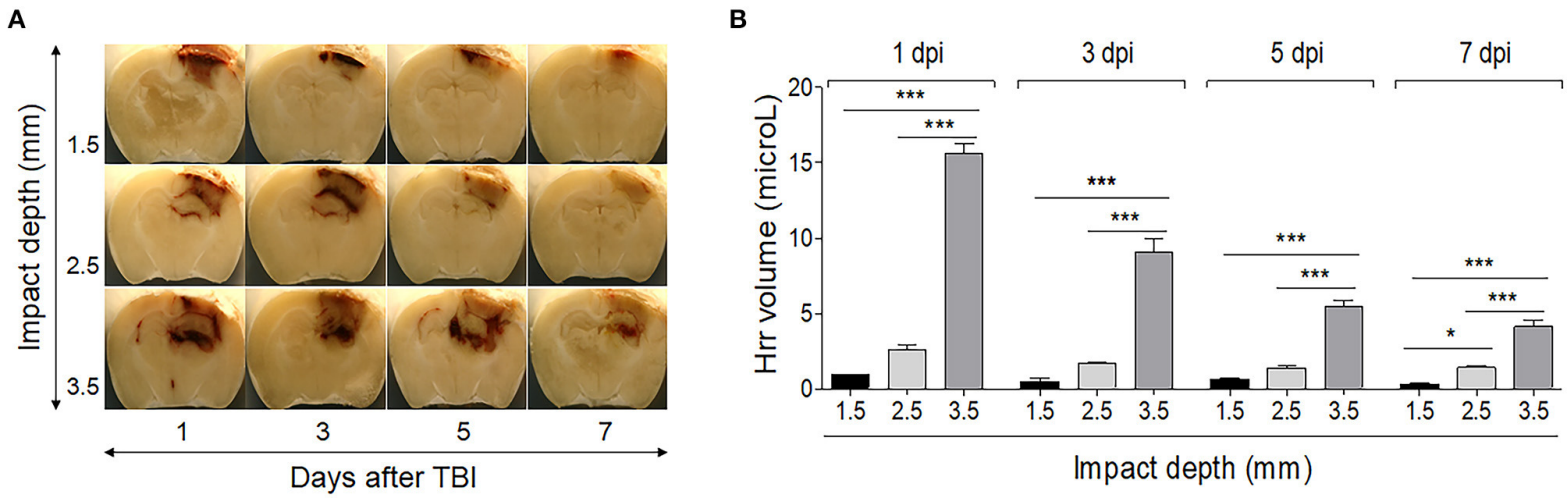
Temporal profile of hemorrhage and hematoma formation in the controlled cortical impact-induced traumatic brain injury mouse model. Photographs of the brain (−1.82 mm from the bregma) were taken using a digital camera during the cryocut process. **(A)** The graph shows the hemorrhage volume in the mouse brain by impacting depth and by day after induction. **(B)** Impacting at 1.5 mm depth produced slight hemorrhage only on the surface of the cortex, while impacting at 2.5 and 3.5 mm produced hemorrhage distributed throughout the cortical area and the hippocampal surrounding ventricle. Values represent means ± SEM (*n* = 6). One-way ANOVA with Tukey's *post-hoc* test; *p < 0.05, ***p < 0.001.

The level of Hrr was related to the severity of the impact. An impact of 1.5-mm depth produced Hrr on the surface of the cortex, while impacts of 2.5 and 3.5 mm produced Hrr distributed throughout the cortical area and the hippocampal surrounding ventricle (Figures 2A,B). The quantity of Hrr in TBI peaked at 1 dpi and gradually decreased until 7 dpi (Figure 2B). Hematoma (mass-like morphology) was observed from 3 dpi and removed during cleaning from 5 dpi. Notably, it was almost cleared in 2.5-mm depth of TBI, while the dark area remained until 7 dpi in 3.5-mm depth of TBI. In addition, following more severe TBI, the brain showed atrophic changes in the hippocampus, which might be due to compression from the space occupied by the hematoma in the upper and lower parts of the hippocampus (Figure 2A).

### Pathological Changes of Warfarin Treatment With/Without Coa VIIa Treatment in Traumatic Brain Injury

**Figure 3:**
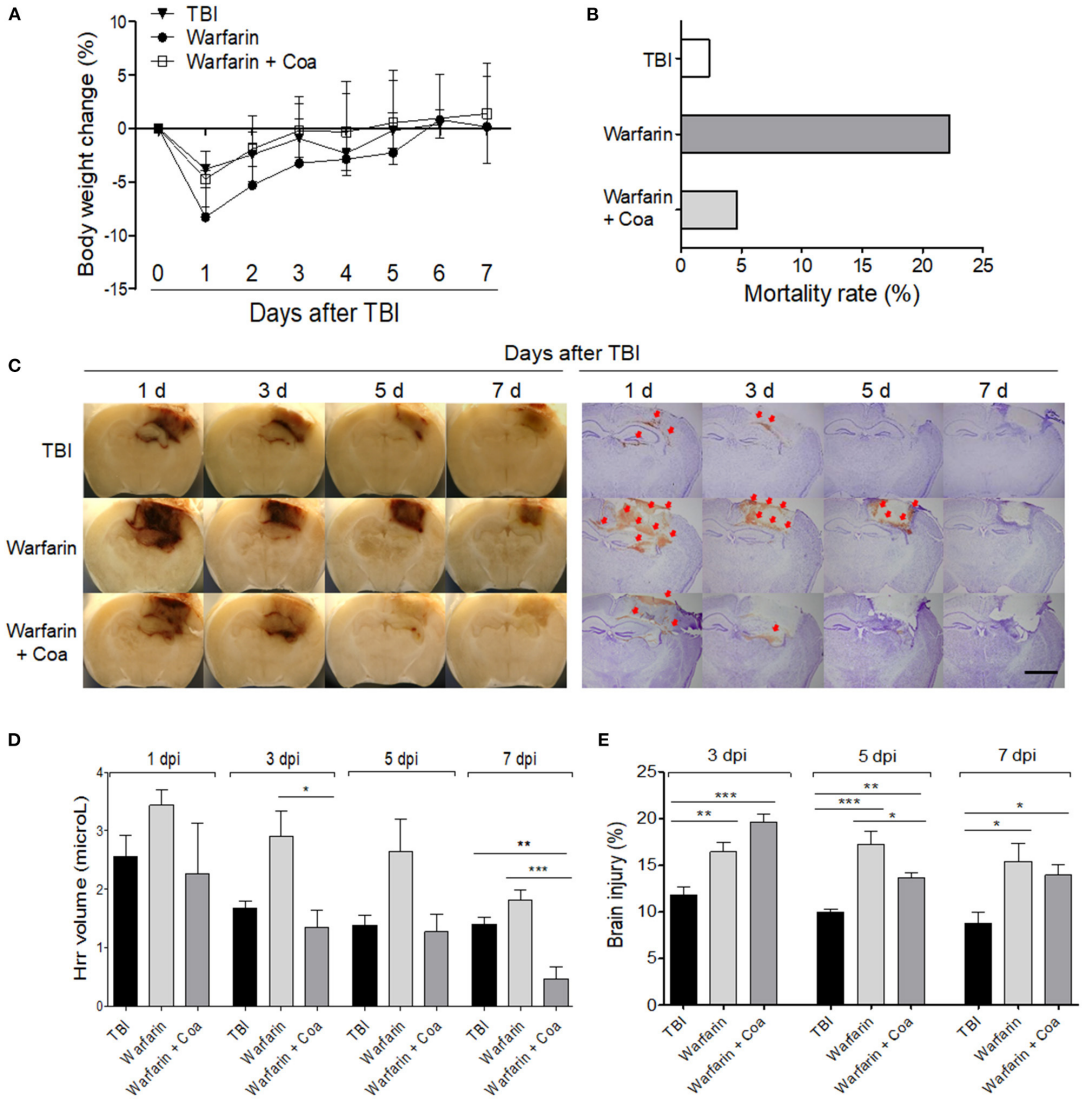
The effect of warfarin and coagulation factor VIIa (Coa) on body weight **(A)** and mortality rate **(B)**, hemorrhage volume **(C,D)**, and brain damage **(C,E)** after TBI. **(C)** Image of a brain sliced and cresyl violet-stained tissue of each group on 1, 3, 5, and 7 days after TBI. The arrows indicate leaked blood. **(D,E)** Graph of the hemorrhage volume (μl) and brain damage (%) of each group, respectively. The scale bar of A is 2 mm. Values represent means ± SEM (*n* = 5–6). One-way ANOVA with Tukey's *post-hoc* test; *p < 0.05, **p < 0.01, ***p < 0.001. TBI, traumatic brain injury.

The body weight of the mice in all the groups decreased immediately after TBI and then increased gradually at 7 dpi. The Warfarin group showed a tendency of body weight loss as compared with the other groups (Figure 3A). The mortality rate was high in the Warfarin group (22.22%), Warfarin + Coa group (4.65%), and TBI (2.32%). The Warfarin + Coa group had a lower mortality rate than the Warfarin group (Figure 3B). The Hrr volume was the highest at 1 dpi and gradually decreased from 3 to 7 dpi. When observed with the naked eye, the Warfarin group showed an increased Hrr volume as compared with the TBI group and a similar pattern to the Hrr volume measurement value (μl). After warfarin pre-treatment, it can be observed visually that Coa VIIa decreases the increased Hrr volume, and Hrr volume measurement value (μl) was significantly reduced (Figures 3C,D). When observed with brain tissue photographs, the amount of Hrr was the highest on day 1 of TBI and gradually decreased until 7 dpi, which was consistent with the hemoglobin content (Figure 3C). On the other hand, the Warfarin group showed an obvious increase in Hrr compared with the TBI group, and the Hrr persisted until 7 dpi. Coa VIIa reduced the Hrr content induced by TBI after warfarin pre-treatment (Figures 3C,D).

TBI induced ~12% of brain damage at 3 dpi and was maintained until 7 dpi, with a slight decrease. Brain damage in the Warfarin group increased by ~17% at 3 dpi and was maintained at over 15% throughout the experiment (*p* < 0.05–0.001 compared with TBI group, Figure 3E). Brain damage in the Warfarin + Coa group increased by ~20% at 1 dpi and decreased rapidly to 13% from 3 dpi to 7 dpi. Coa VIIa decreased the warfarin-induced worsening of brain damage at 5 dpi (*p* < 0.05 compared with Warfarin group, Figure 3E).

### IBA-1 Expression at 7 dpi

**Figure 4:**
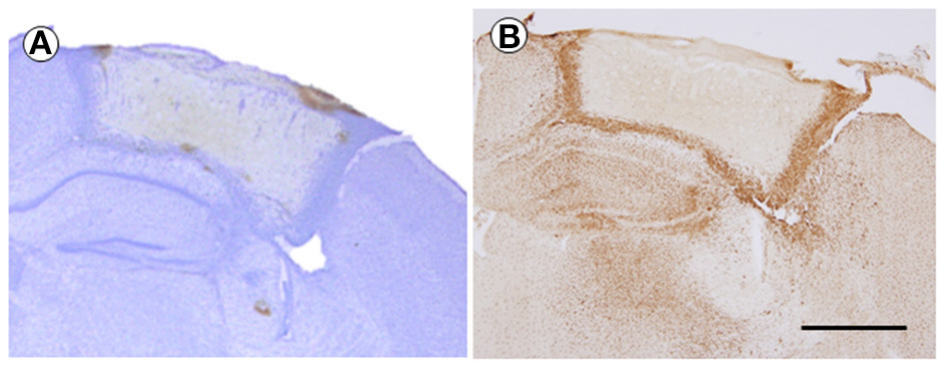
The cresyl violet (CV)-stained brain **(A)** and immunohistochemistry of IBA-1 **(B)** at 7 days after TBI (2.5 mm of depth). The brain sections were from the same mouse. The magnification was ×40, and the scale bar is 1 mm. TBI, traumatic brain injury.

CV-stained tissue showed the differences of density among injury-core, boundary area, and intact area in TBI (2.5-mm depth, 7 dpi). The boundary area that showed high density was showed as boundary between injury and intact area (Figure 4A). IBA-1-positive cells were observed, which also formed the border between the injured and uninjured areas by forming boundary area, which was similar with CV-stained brain and also found in the hippocampal area and the thalamic area. In the comparison between CV and IBA-1-stained tissues, it was found that the dense area of the CV-stained tissues was consistent with the IBA-1-stained tissues. Notably, any background color was invisible in the injury-core region of both stained brains (Figure 4B).

## Discussion

In this study, we presented a new protocol for a TBI mouse model that allows the study of pathological events surrounding the injury-core, including Hrr, hematoma, and the damage itself. In terms of Hrr and hematoma, the current results showed that the amount and distribution of Hrr varied depending on the severity of the brain injury over time. In order to further verify the utility of the model, as a result of comparing the amount of Hrr after administration of the hemodynamic agents, it was found that the amount of Hrr increased in the warfarin-treated group, and that Coa VIIa reversed the Hrr caused by warfarin.

Many studies using the CCI-TBI model have not investigated the features of Hrr, hematoma, and injury-core. This is because of the difficulty in treating the injury-core during the procedure of histology. This study solved this problem by preserving the area up to 7 dpi via a slight minor modification of the histological procedure: first, post-fixation of the mouse head for 24 h for hardening (fixing) of the injury-core; and second, performing the histological process, including brain isolation, cutting the brain slice, and staining with the ipsilateral skull bone remaining attached to the brain. We found that the skull of the mouse was soft and flexible enough to be cut cleanly using a cryocut and that the injury-core was well-preserved when the brain was carefully cut with the skull remaining. However, the rat skull is harder than the mouse skull, and it is therefore difficult to perform the current protocol.

The current results of a typical Hrr pattern depending on severity over time showed that the Hrr reached its peak from 1 dpi; the color was fresh red without the mass-like morphology. The leaked blood volume decreased slightly at 3 dpi, resulting in a dark red, shape-like mass of blood. The blood distribution of mild damage was located only on the surface of the parietal cortex, while that of moderate damage was located at the upper surface of the hippocampus along the cortex. In addition, severe damage produced blood distribution even in the third ventricle along the entire affected cortex and hippocampal area (Figure 2A). Thus, it is suggested that the current method might be suitable for Hrr and hematoma studies using CCI-TBI, and the mild and moderate types should be studied before 3–5 days, and the severe type should be performed from 1 day.

To further validate the utility of the model, a 2.5-mm depth impacting suitable for investigating Hrr changes and distribution with low mortality was selected. For CCI-TBI induction, the impact depth is closely associated with the severity of damage; however, impact depth at 1 mm did not induce obvious Hrr in our system, which was the reason for the differences in severity and impact depth as compared with those used in other studies (1.5, 2.5, and 3.5–4 mm) (18). In TBI mouse (2.5-mm depth), warfarin induced body weight loss, massive increase in Hrr volume, brain damage, and mortality rate. This was reversed by the administration of an anticoagulation factor. Warfarin is an anticoagulant and has been widely used worldwide, especially in intravascular coagulation and coagulation disorders (19). Previous studies found that when a patient pre-treated with warfarin sustains brain injury due to traumatic impact, such as a traffic accident, the side effect is Hrr to death (20). To date, no time course of warfarin-induced hemorrhagic changes >3 days in TBI has been reported; endogenous plasminogen activators mediate progressive intracerebral Hrr after TBI in mice (16, 21). We therefore propose that the protocol might also be suitable for studying the side effects, including excessive Hrr of warfarin in TBI. Several methods have been proposed for the side effects of warfarin in TBI (22). However, there are few studies on the side effects of warfarin, especially Hrr control, through animal studies. This may be due to the limitation of the existing model protocol mentioned above. Therefore, to confirm that this protocol is a solution to this, we investigated the effect of the hemodynamic agent Coa VIIa to determine whether it could suppress the excessive Hrr pathology caused by warfarin. Coa VIIa is known to increase coagulation by directly activating factor X on the surface of activated platelets (23). It was also reported in previous studies that Coa VIIa was effective in reversing warfarin anticoagulation and reducing intracranial Hrr, but it did not investigate the effects at >1 dpi (24). The current results have enabled studies up to 7 dpi and show that FVIIa inhibits warfarin-induced high mortality rate, Hrr at 3 and 7 dpi, and brain damage at 5 dpi in a TBI mouse model. To the best of our knowledge, this is the first study to demonstrate the exacerbation of warfarin-induced hemorrhagic brain injury in TBI and the inhibitory effect of Coa VIIa as a treatment using an animal model.

In the current study, we evaluated the tissue condition by immunohistochemistry using IBA-a, an active microglia marker. The current result showed that IBA-1-positive cells obviously formed a boundary area of the injury-core at 7 dpi. The injury-core was visible clearly without any IBA-1-positive cells or background color (false-positive expression). The current result suggests that IBA-1-positive cells might be associated with action around injury and that the immunohistochemistry using the TBI brain tissue prepared by the current protocol worked well with the advantage of being able to observe both the injury-core and penumbral region.

To date, it has been difficult to study the temporal profile of Hrr, hematoma, and brain injury itself. This is because there were areas for improvement related to the injury-core during the course of the experiment. We modified the existing protocol (histopathological steps) to preserve the injury-core and successfully elicited the temporal profiles of Hrr depending on the severity of injury and hemodynamic intervention until 7 dpi. In total, the current modified method might be useful in research using the CCI-TBI model for conducting studies on hematoma, Hrr, and injury-core. However, there is a limitation of the current protocol that it does not reflect the clinical condition such as craniotomy to lower the ICP elevation due to Hrr in TBI patients. Further studies are needed to investigate the relationship between leaked blood and neuronal death, and the pathological changes in hematoma resolution using CCI-TBI mice model and to establish the optimal animal model based on clinical situation in human TBI patients.

## Data Availability Statement

The original contributions presented in the study are included in the article/supplementary material, further inquiries can be directed to the corresponding author/s.

## Ethics Statement

The animal study was reviewed and approved by Kyung Hee University Institutional Animal Care and Use Committee (KHUASP(SE)-20-132).

## Author Contributions

YB, BDL, and HJ contributed to conception and design of the study. JB, B-JL, and J-WP organized the database. HJ and YB wrote the first draft of the manuscript. HJ, YB, and BDL wrote sections of the manuscript. All authors contributed to manuscript revision, read, and approved the submitted version.

## Funding

This work was supported by the National Research Foundation of Korea (NRF) grant funded by the Korea government (MSIT) (No. 2020R1A2C1008603).

## Conflict of Interest

The authors declare that the research was conducted in the absence of any commercial or financial relationships that could be construed as a potential conflict of interest.

## Publisher's Note

All claims expressed in this article are solely those of the authors and do not necessarily represent those of their affiliated organizations, or those of the publisher, the editors and the reviewers. Any product that may be evaluated in this article, or claim that may be made by its manufacturer, is not guaranteed or endorsed by the publisher.
